# Willingness to participate in COVID-19 vaccine trials; a survey among a population of healthcare workers in Uganda

**DOI:** 10.1371/journal.pone.0251992

**Published:** 2021-05-27

**Authors:** Jonathan Kitonsa, Onesmus Kamacooko, Ubaldo Mushabe Bahemuka, Freddie Kibengo, Ayoub Kakande, Anne Wajja, Vincent Basajja, Alfred Lumala, Edward Ssemwanga, Robert Asaba, Joseph Mugisha, Benjamin F. Pierce, Robin Shattock, Pontiano Kaleebu, Eugene Ruzagira

**Affiliations:** 1 Medical Research Council / Uganda Virus Research Institute & London School of Hygiene and Tropical Medicine Uganda Research Unit, Entebbe, Uganda; 2 Kitovu Hospital, Masaka District, Uganda; 3 Villa Maria Hospital, Kalungu District, Uganda; 4 Our Lady of Consolata Kisubi Hospital, Wakiso District, Uganda; 5 Department of Infectious Disease, Imperial College London, Norfolk Place, London, United Kingdom; University of Toronto, CANADA

## Abstract

**Background:**

Healthcare workers (HCWs) are at high risk of acquiring SARS-CoV-2 and COVID-19 and may therefore be a suitable population for COVID-19 vaccine trials. We conducted a survey to evaluate willingness-to-participate in COVID-19 vaccine trials in a population of HCWs at three hospitals in Uganda.

**Methods:**

The survey was conducted between September and November 2020. Using a standardised questionnaire, data were collected on socio-demographics, previous participation in health research, COVID-19 information sources, underlying health conditions, and willingness-to-participate in COVID-19 vaccine trials. Data were analysed descriptively and a binomial generalised linear model with a log link function used to investigate factors associated with unwillingness to participate.

**Results:**

657 HCWs (female, 63%) were enrolled with a mean age of 33 years (Standard Deviation, 10). Overall willingness-to-participate was 70.2%. Key motivating factors for participation were: hope of being protected against COVID-19 (81.1%), altruism (73.3%), and the opportunity to get health care (26.0%). Selected hypothetical trial attributes reduced willingness-to-participate as follows: weekly-quarterly study visits over a 12-month period (70.2%-63.2%, *P* = 0.026); provision of approximately 50ml of blood at each study visit (70.2%-63.2%, *P* = 0.026); risk of mild-moderate local adverse reactions (70.2%-60.3%, *P*<0.001); chance of receiving candidate vaccine or placebo (70.2%-56.9%, *P*<0.001); and delay of pregnancy [Overall, 70.2%-57.1% *P*<0.001); Female, 62.8%-48.4% (*P =* 0.002); Male, 82.5%-71.5% (*P =* 0.003)]. Collectively, these attributes reduced willingness-to-participate from [70.2%-42.2% (*P*<0.001) overall; 82.5%-58.1% (*P*<0.001) in men; 62.8%-32.6% (*P<0*.*001*) in women]. Among individuals that were unwilling to participate, the commonest barriers were concerns over vaccine safety (54.6%) and fear of catching SARS-CoV-2 (31.6%). Unwillingness to participate was associated with being female (aRR 1.97, CI 1.46–2.67, *P*<0.001) and having university or other higher-level education (aRR 1.52, CI 1.05–2.2, *P* = 0.026).

**Conclusions:**

Willingness-to-participate in COVID-19 vaccine trials among HCWs in Uganda is high but may be affected by vaccine trial requirements and concerns about the safety of candidate vaccines.

## Introduction

The COVID-19 pandemic that started in December 2019 has continued to cause devastating effects globally. Approximately 122,500,000 cases and 2,700,000 deaths had been reported globally by 21 March 2021 [1]. The pandemic has also had negative effects on other sectors including economies, social aspects of life, among others, some of these happening due to control strategies such as lockdowns and restriction of business and trade [2, 3]. Several therapies have been tested for use in the management of COVID-19 mostly through repurposing, with most of these showing either no or low efficacy [4–6].

The best solution to help control COVID-19 will most likely be vaccines with adequate efficacy and effectiveness, and the development and deployment of these remains a priority [7–9]. Many vaccines have progressed through different phases of development with some demonstrating good safety and immunogenicity profiles [9], while others have demonstrated very good efficacy. Pfizer-BioNTech and Moderna’s messenger ribonucleic acid (mRNA)-based vaccines have both shown efficacy at 95%; Oxford–AstraZeneca’s ChAdOx1 nCoV-19 and Johnson and Johnson’s adenovirus based vaccines have demonstrated efficacies of 70% and 66% respectively [10–13]. These four vaccines have received emergency use authorization by the United States Food and Drug Administration, European Medicines Agency, and/or the World Health Organisation [14–16]. Other vaccines such as Russia’s sputnik V vaccine and Novavax’s NVX-CoV2373 vaccine have also shown very good efficacy [17, 18]. While the progress made in bringing on board new vaccines is encouraging, the emergence of SARS-CoV-2 variants so far reported in Brazil, the United Kingdom, and South Africa may cause new setbacks. For example, the South African variant (501Y.V2) appears to reduce the efficacy of Oxford–AstraZeneca’s ChAdOx1 nCoV-19 vaccine to as low as 10% [19]. This variant has already been identified in several African countries including Uganda [1] where the Oxford–AstraZeneca vaccine is currently being rolled out. It is therefore necessary that efforts to develop and test new COVID-19 vaccines continue in order to cater for the global unmet need as well as protect against new variants of the virus.

In addition, the available vaccine candidates have been investigated mostly in the USA, Europe, China, and South America with minimal participation of African populations [10]. There is a possibility that COVID-19 vaccines may have different pharmacokinetics, pharmacodynamics, and other outcomes among the African population. Previous studies have shown that genetics, racial background, and the environment can affect response to vaccines [20–22]. It is therefore imperative that vaccines against COVID-19 are investigated amongst African populations.

Globally, healthcare workers (HCWs) are among the population groups at highest risk of acquiring SARS-CoV-2 infection and developing COVID-19 disease due to their exposure to patients or infectious material at work as well as in the community [23]. At the end of October 2020, the International Council of Nurses confirmed that 1,500 nurses had died from COVID-19 in 44 countries and estimated healthcare worker COVID-19 fatalities to be more than 20,000 worldwide [24]. In Africa, HCWs may be particularly vulnerable to contracting SARS-CoV-2 infection from patients who have subclinical or asymptomatic infection, those who may present with fever related to other infections but who also have SARS-CoV-2 infection, and from super-spreading events that may not be easily investigated and controlled [25]. Moreover, the limited critical care infrastructure in many parts of Africa may lead to high mortality rates among HCWs who become infected with SARS-CoV-2 [25]. As of 25 March 2021, HCWs constituted 4.7% of the 40,767 confirmed COVID-19 cases in Uganda [26].

It is however not clear if HCWs from Africa would be willing to participate in COVID-19 vaccine trials. This is partly because the pandemic has been shrouded with misinformation, creating negative impressions about potential vaccines against COVID-19 [27, 28]. Most studies that have evaluated willingness-to-participate in COVID-19 vaccine trials that we are aware of have been done outside Africa and have not targeted HCWs [29, 30]. We therefore conducted a study to assess willingness-to-participate in COVID-19 vaccine trials and associated factors among HCWs in Uganda.

## Methods

### Study design and setting

This was a cross-sectional study conducted between September 2020 and November 2020, using a face-to-face interviewer-administered electronic questionnaire. The study was conducted by the Medical Research Council/Uganda Virus Research Institute and London School of Hygiene and Tropical Medicine (MRC/UVRI and LSHTM) Uganda Research Unit in collaboration with three Private Not for Profit (PNFP) faith-based hospitals, with two of these found in southwest Uganda and one in central Uganda: i) Kitovu Hospital, a 248-bed hospital located in Masaka city, about 140km from Uganda’s capital, Kampala; ii) Villa Maria Hospital, a 100-bed hospital located in a rural setting in Villa Maria, Kalungu District, about 15km from Masaka city; iii) Our lady of Consolata Kisubi Hospital, a 110-bed hospital located in Kisubi, Wakiso district, approximately 28km from Kampala. These hospitals were not gazetted to manage COVID-19 patients by the time the study was conducted. They were purposely selected in order to have a population of HCWs that had limited or no experience in managing COVID-19 patients. Other hospitals in these settings such as Masaka Regional Referral Hospital and Entebbe Grade B Hospital were not included because they were gazetted to manage COVID-19 and staff had received extensive training related to this.

### Enrolment of study participants

Participation was open to all HCWs at the three hospitals including health professionals providing direct medical care to patients, hereby referred to as medical HCWs (medical doctors, nurses, nursing assistants, allied health professionals, medical/nursing students, and other roles involving direct patient care) and those providing support services, hereby referred to as non-medical HCWs (administrators, receptionists, cleaners, janitors, drivers, etc.). All enrolment procedures were conducted at the hospitals by research assistants. Before participant enrolment, the study investigators held meetings with the administrators of the three hospitals to confirm their interest in the study, establish contact persons for the research team, confirm numbers of and obtain lists of the HCWs, and agree on the procedures for approaching staff. Hospital administrators were requested to inform their staff about the planned study. With the assistance of the designated hospital contact persons, research assistants organised and conducted seminars for groups of 20–30 HCWs at which they provided detailed information about the study and answered attendees’ questions. At the end of each session, research assistants scheduled one-on-one sessions for individuals who expressed interest in the study. In the one-on-one sessions, research assistants provided detailed information about the study, answered participants’ questions, and conducted eligibility assessments. Individuals were enrolled in the study if they were aged ≥18 years, willing and able to provide written informed consent, willing to complete an interviewer-administered electronic questionnaire, and not confirmed or suspected to have COVID-19.

### Data collection and study variables

A predesigned standardised electronic questionnaire was administered by experienced research assistants using encrypted computer tablets on the same day of obtaining informed consent. Due to the paucity of context-specific data on COVID-19 vaccine research at the time of designing this study, questionnaire items were majorly informed by findings from previous research on willingness-to-participate in HIV vaccine trials [31, 32].

Independent variables included socio-demographic characteristics including age, sex, marital status, occupation, role/position at the health facility, highest level of education, household size; previous participation in health research; source of information on COVID-19; presence of underlying health conditions.

The dependent variable was willingness-to-participate in future COVID-19 vaccine trials. To evaluate this, participants were asked if they would be willing to participate in COVID-19 vaccine trials with possible responses being “definitely yes”, “probably yes”, “definitely no”, “probably no” and “Not sure”. Participants who stated that they were willing to participate (“definitely yes”, “probably yes”) in COVID-19 vaccine trials were asked to state what motivated them (altruism, hope of being protected against COVID-19, opportunity to get health care, monetary gain, etc.). Additionally, these participants were asked if they would still be willing to participate in a COVID-19 vaccine trial given the following hypothetical trial attributes: a trial schedule requiring attendance of frequent (weekly-quarterly) clinic visits over a 12-month period, provision of blood samples (approximately 50ml) at each of the clinic visits, the possibility of receiving either the candidate vaccine or placebo, the risk of experiencing mild-moderate reactions at the injection site; and willingness to avoid/delay pregnancy by using effective contraception if female or to avoid impregnating a female partner, if male, during the trial period. Participants who stated that they were unwilling to participate in COVID-19 vaccine trials (“definitely no”, “probably no” and “not sure”) were asked to state what their concerns/barriers were? Possible responses included anxiety/fear of catching SARS-CoV-2, concerns over safety of vaccines, fear of injections, fear of being stigmatised, lack of time, and other concerns.

### Sample size considerations

We adopted the sample size formula for cross-sectional surveys [33] to estimate the precision (half-width of 95% CI) around the estimated proportion of willingness to participate for different values (10% to 90%) of the true population proportion and varying sample size. We estimated that even a minimum sample size of 450 participants would provide an estimate of willingness-to-participate in COVID-19 vaccine trials with a 95% confidence interval width of no more than 5 percentage points.

### Statistical analysis

Data from computer tablets was uploaded onto a REDCap database and exported to STATA (College Station, TX, version 15.0) for analysis. Descriptive statistics such as frequencies and percentages were used to summarise categorical variables, while continuous variables were summarised using means (standard deviations) and/or medians (interquartile ranges). Willingness-to-participate was estimated as the sum of all participants whose responses were “definitely yes” and “probably yes” divided by the total number of interviewed participants expressed as a percentage. Participants whose responses were “definitely no”, “probably no” and “not sure” were categorised as unwilling to participate.

McNemar’s chi-square test was used to evaluate the impact of different hypothetical trial attributes on willingness-to-participate. Due to a high proportion of willingness-to-participate, a binomial generalised linear model with a log link function was used to investigate factors associated with unwillingness to participate. Only factors that were significantly associated with unwillingness to participate at the 15% level using a likelihood ratio test (LRT) were considered for the multivariable model [23]. Factors were removed from the multivariable model if doing so did not make the fit of the model significantly worse at the 5% level. A *P* value <0.05 was considered statistically significant. Results are presented as adjusted relative risks (aRR) with 95% confidence intervals (CI).

### Ethics approval and consent to participate

The study protocol and informed consent documents were reviewed and approved by the Uganda Virus Research Institute Research Ethics Committee (Ref: GC/127/20/07/780), the Uganda National Council for Science and Technology (Ref: HS807ES), and The London School of Hygiene and Tropical Medicine Research Ethics Committee (Ref: 22602). All participant interviews were conducted after obtaining written informed consent.

## Results

### Participant characteristics

All 717 HCWs at the three hospitals were invited to participate in the study and of these, 657 (91.6%) agreed to participate and were enrolled. Among those enrolled, the mean age (Standard Deviation) was 33 (10) years with most being female (62.6%), married (55.6%), medical HCWs (64.7%), and educated to university or other high-level institution (76.8%). The commonest sources of information on COVID-19 were news media e.g., radio and TV (96.0%) followed by social media platforms (70.2%).

### Willingness-to-participate in COVID-19 vaccine trials and impact of trial attributes

Overall, 461 (70.2%) participants expressed willingness-to-participate in COVID-19 vaccine trials. Motivators for willingness-to-participate included hope of being protected against COVID-19 (81.1%), altruism (73.3%), the opportunity to get health care (26.0%), and the hope of receiving monetary benefits (8.5%).

The impact of hypothetical vaccine trial attributes on willingness-to-participate is summarised in Table 1. In general these attributes reduced willingness-to-participate as follows: requirement to attend frequent (weekly-quarterly) clinic visits over a 12-month period [Overall, 70.2–63.2% (McNemar’s χ^2^ = 4.95; *P* = 0.026); Men, 82.5%-73.6% (McNemar’s χ^2^ = 12.50; *P* = 0.026); Women, 62.8%-56.9% (McNemar’s χ^2^ = 1.67; *P* = 0.182)]; requirement to provide blood samples (approximately 50ml) at each of the clinic visits [Overall, 70.2%-63.2% (McNemar’s χ^2^ = 4.95; *P* = 0.026); Men, 82.5%-78.5% (McNemar’s χ^2^ = 1.38; *P* = 0.292); Women 62.8%-54.0% (McNemar’s χ^2^ = 1.98; *P* = 0.159); risk of experiencing mild to moderate adverse events at the injection site [Overall, 70.2%-60.3% (McNemar’s χ^2^ = 10.50; *P*<0.001); Men, 82.5%-74.4% (McNemar’s χ^2^ = 6.55; *P* = 0.042); Women, 62.8%-46.5% McNemar’s χ^2^ = 28.30; *P* = 0.<001]; possibility of receiving either the candidate vaccine or placebo [Overall, 70.2%-56.9% (McNemar’s χ^2^ = 37.20; *P*<0.001); Men, 82.5%-74.4% (McNemar’s χ^2^ = 9.00; *P* = 0.003); women, 62.8%-46.5% (McNemar’s χ^2^ = 28.30; *P* = <0.001)]; requirement to avoid pregnancy/ impregnating a female partner [Overall, 70.2%-57.1% (McNemar’s χ^2^ = 21.50; *P<*0.001); Men, 82.5%-71.5% (McNemar’s χ^2^ = 9.00; *P* = 0.003); Women, 62.8%-48.4% (McNemar’s χ^2^ = 12.80; *P* = 0.002)]. Collectively, these trial attributes reduced willingness-to-participate as follows [Overall, 70.2%-42.2%, (McNemar’s χ^2^ = 164.00; *P*<0.001); Men, 82.5%-58.1%, (McNemar’s χ^2^ = 60.00; *P*<0.001); Men, 62.8%-32.6%, (McNemar’s χ^2^ = 104.50; *P*<0.001)].

**Table 1:** Impact of trial attributes on willingness-to-participate in COVID-19 vaccine trials (overall and by sex).

Hypothetical trial attribute	Overall (n = 657)			Men (n = 246)			Women (n = 411)		
	Proportion willing to participate in a trial without the hypothetical attribute (%)	Proportion still willing to participate in a trial with the hypothetical attribute (%)	McNemar’s chi-square value; *P*-value	Proportion willing to participate in a trial without the hypothetical attribute (%)	Proportion still willing to participate in a trial with the hypothetical attribute (%)	McNemar’s chi-square value; *P*-value	Proportion willing to participate in a trial without the hypothetical attribute (%)	Proportion still willing to participate in a trial with the hypothetical attribute (%)	McNemar’s chi-square value; *P*-value
Attendance of frequent (weekly-quarterly) clinic visits over a 12-month period	70.2	63.2	4.95; 0.026	82.5	73.6	12.50; 0.026	62.8	56.9	1.67; 0.182
Provision of blood samples (approximately 50ml) at each of the clinic visits	70.2	63.2	4.95; 0.026	82.5	78.5	1.38; 0.292	62.8	54.0	1.98; 0.159
Risk of experiencing mild to moderate adverse events at the injection site	70.2	60.3	10.50; <0.001	82.5	75.6	6.55; 0.011	62.8	51.1	5.26; 0.022
Receipt of either the candidate vaccine or placebo	70.2	56.9	37.20; <0.001	82.5	74.4	9.00; 0.003	62.8	46.5	28.30; <0.001
Requirement to avoid pregnancy/impregnating female partner (Overall)	70.2	57.1	21.50; <0.001	-	-	-	-	-	-
Requirement to avoid pregnancy/impregnating female partner (Men)	82.5	-	-	82.5	71.5	9.00; 0.003	-	-	-
Requirement to avoid pregnancy/impregnating female partner (women)	62.8	-	-	-	-	-	62.8	48.4	12.80; 0.002
All attributes of the trial	70.2	42.2	164.00; <0.001	82.5	58.1	60.00; <0.001	62.8	32.6	104.50;<0.001

### Unwillingness-to-participate in COVID-19 vaccine trials

Reasons for unwillingness to participate in COVID-19 vaccine trials included concerns about the safety of candidate vaccines (54.6%), anxiety/fear of catching SARS-CoV-2 (31.6%), fear of being stigmatised (8.0%), fear of injections (4%), and inability to commit time (4.7%). Table 2 provides a summary of the relationship between selected factors and unwillingness to participate in COVID-19 vaccine trials. Unwillingness to participate was significantly associated with being female (aRR 1.96, CI 1.45–2.66, *P*<0.001); having university or other higher-level education (aRR 1.50, CI 1.04–2.16, *P* = 0.030); and marginally associated with use of traditional news media (Television, Radios, Magazines, Newspapers) as source of information on COVID-19 (aRR 0.66, CI 0.43–1.00, *P* = 0.050) and presence of underlying health conditions (aRR 1.29, CI 0.98–1.69, *P* = 0.070). Age, marital status, occupation, and use of social media as source of information were not significantly associated with unwillingness to participate in COVID-19 vaccine trials.

**Table 2:** Factors associated with unwillingness to participate in COVID-19 vaccine trials.

Characteristic	Level	N (col %)	Unwillingness n (row %)	uRR (95% CI)	LRT *P*-value	aRR (95% CI)	*P*-value
**Overall**		657 (100)	196 (29.8)				
**Age group (years)**	18–25	160 (24.4)	54 (33.8)	Ref	0.043	Ref	
	26–35	290 (44.1)	72 (24.8)	0.74 (0.55–0.98)		0.85 (0.64–1.13)	0.260
	>35	207 (31.5)	70 (33.8)	1.01 (0.75–1.34)		1.10 (0.82–1.47)	0.530
**Sex**	Male	246 (37.4)	43 (17.5)	Ref	<0.001	Ref	
	Female	411 (62.6)	153 (37.2)	2.13 (1.58–2.87)		1.97 (1.46–2.67)	<0.001
**Marital Status**	Not married	292 (44.4)	93 (31.9)	Ref	0.313		
	Married	365 (55.6)	103 (28.2)	0.87 (0.70–1.12)			
**Occupation**	Non-medical HCW	232 (35.3)	55 (23.7)	Ref	0.010	Ref	
	Medical HCW	425 (64.7)	141 (33.2)	1.40 (1.07–1.83)		1.11 (0.83–1.49)	0.480
**Highest education level**	≤A-level	152 (23.1)	30 (19.7)	Ref	<0.001	Ref	
	University/other higher-level institution	505 (76.9)	166 (32.9)	1.67 (1.18–2.35)		1.52 (1.05–2.20)	0.030
**Household size**	1–2 persons	255 (38.8)	82 (32.2)	Ref	0.360		
	3–5 persons	255 (38.8)	68 (26.7)	0.83 (0.63–1.09)			
	>5 persons	147 (22,4)	46 (31.3)	0.97 (0.72–1.31)			
**Previously volunteered to participate in health research**	No	522 (79.5)	161 (30.8)	Ref	0.261		
	Yes	135 (20.5)	35 (25.9)	0.84 (0.61–1.15)			
**Presence of underlying chronic health condition**	No	564 (85.8)	159 (28.2)	Ref	0.017	Ref	
	Yes	93 (14.2)	37 (39.8)	1.41 (1.06–1.87)		1.29 (0.98–1.69)	0.070
Use traditional news media as source of information on COVID-19*	No	26 (4)	11 (42.3)	Ref	0.122		
	Yes	631 (96)	185 (29.3)	0.69 (0.44–1.10)		0.66 (0.43–1.00)	0.050
**Use social media as source of information**	No	196 (29.8)	61 (31.1)	Ref	0.636		
	Yes	461 (70.2)	135 (29.3)	0.94 (0.73–1.21)			

uRR, Unadjusted odds ratio; aRR, Adjusted odds ratio; CI, Confidence interval; LRT, Log likelihood ratio test; Ref, Reference

*Television, Radios, Magazines, Newspapers.

## Discussion

We found a high level (70.2%) of willingness-to-participate in COVID-19 vaccine trials in this cohort of HCWs in Uganda. Willingness-to-participate in COVID-19 vaccine trials was slightly lower in a cohort of young adults in China at 64% [29], and much lower in an online survey conducted among adults in the general population in France (48%) [30]. In the latter study, being a HCW was positively associated with willingness-to-participate in COVID-19 vaccine trials. HCWs may perceive themselves to be at higher risk of contracting SARS-CoV-2 than other persons given their exposure, and therefore may be more willing to participate in research to find a COVID-19 vaccine. Detoc et al showed that individuals who perceived themselves to be at increased risk of acquiring COVID-19 were more willing to participate in a vaccine trial [30].

Consistent with previous studies [30, 31], we found that unwillingness to participate was significantly associated with sex, with female participants more unwilling to participate than their male counterparts. In general, women are less likely to participate in clinical trials [34]. Reasons for this may include time limitations due to family and work obligations, the need to consult or k permission from male partners, relatives, or even community members before they can participate, and concerns about contraceptive use and future fertility [34, 35]. Despite these difficulties, efforts should be made to recruit women in COVID-19 vaccine trials since there may be sex-based differences in vaccine efficacy and safety outcomes [36].

We found that individuals with university or other higher-level education had significantly higher prevalence of unwillingness to participate in COVID-19 vaccine trials compared to those with lower education. High education levels have been found to be associated with decreased willingness to participate in vaccine research elsewhere [37]. Participants with high levels of education are less likely to be motivated by non-altruistic reasons [38], a factor that may contribute to the relatively high level of unwillingness to participate in this sub-population. A high level of education may also be associated with having more knowledge about clinical trials. People with more knowledge about clinical trials and the adverse events that may occur when one receives an intervention have previously been found to be less willing to participate in trials [39].

Unwillingness to participate was marginally lower among participants who depended on traditional news media (Television, Radio, Magazines, Newspapers) for information on COVID-19 compared to those that did not. The use of traditional media has been associated with improved knowledge about COVID-19 [40–42] which may be critical in influencing support for research on strategies to control COVID-19. Unwillingness to participate was marginally higher among individuals who reported having underlying health conditions compared to those that did not. This is probably because participants with underlying health conditions had concerns about the possibility of experiencing frequent or severe vaccine adverse events.

Hope of being protected against COVID-19 was the commonest motivator for willingness-to-participate in COVID-19 vaccine trials. This is most likely because of the misconception, reported in previous studies that candidate vaccines can offer protection against infection [31, 32]. This phenomenon, referred to as “preventive misconception” [43], is characterised by trial participants overestimating the protection they may receive when they take part in a prevention trial. This may have various implications such as trial participants not practicing adequate control measures with the assumption that they are “already safe” [44]. The second most reported motivator for willingness-to-participate was altruism. This is consistent with literature which shows that many people enrol in trials for altruistic reasons and to contribute to scientific research [45–47]. In the case of COVID-19, people might be willing to support research that can help identify a solution due to its devastating impact on millions of people worldwide. Consistent with previous studies in settings with limited access to high-quality healthcare [31, 48], we found that the opportunity to get healthcare services is a motivator for participating in clinical trials for a considerable number of the study participants. Few participants cited the possibility of receiving monetary payments as a primary motivator for willingness-to-participate. This is reassuring as research participants who are primarily motivated by payment may not be in position to properly assess the risks posed by research studies which presents ethical difficulties [49].

Different hypothetical trial attributes reduced willingness-to-participate in COVID-19 vaccine trials. The requirement to avoid/delay pregnancy significantly reduced willingness-to-participate with the largest impact observed among women. This finding has been reported in previous studies [31, 32] and may be related to a reluctance to use effective contraception and concerns about future fertility [35]. Investigators for COVID-19 vaccine trials in this and similar settings will need to address barriers to contraceptive use particularly among women.

The possibility to receive either the candidate vaccine or placebo reduced willingness-to-participate in COVID-19 vaccine trials with the largest impact observed among women. This is not surprising since hope of being protected from COVID-19 was a major motivation for willingness-to-participate in this study. This calls for clear education of trial participants before and during actual COVID-19 vaccine trials, emphasising that candidate vaccines are experimental and may not provide protection, and that inclusion of a placebo arm is a crucial methodological aspect of clinical trials.

We found that the possibility to experience mild to moderate short-lived adverse events at the injection site significantly reduced willingness-to-participate in COVID-19 vaccine trials. Also, concerns over the safety of vaccines in general and fear of catching SARS-CoV-2 from the vaccine were major reasons for unwillingness to participate. Safety concerns are common barriers to vaccine trial participation and have been cited in several studies [29, 31, 32, 37, 50]. In the case of COVID-19 vaccine trials, these concerns may have been enhanced since COVID-19 vaccine trials have occasionally been paused due to safety concerns, which occurrences have been extensively publicised through the press and social media. As part of preparation for COVID-19 vaccine trials, it is important that potential study communities and participants are provided detailed information on the possible side effects of candidate vaccines and more importantly, reassured about the rigour applied to safety monitoring in trials.

Willingness-to-participate reduced significantly in men but not women for a trial that would require participants to attend frequent (weekly-quarterly) study visits over a 12-month period. This signals possible concerns about time commitments that will be required of participants in planned COVID-19 vaccine trials particularly those that aim to recruit individuals such as HCWs with busy and demanding work schedules. The differential impact between men and women may be due to differences in willingness or ability to take time off work. HCWs might be expected to have a good understanding of the rationale for obtaining blood samples from participants in clinical trials. Nevertheless, we observed that the requirement to provide approximately 50ml of blood at each study visit reduced willingness-to-participate particularly among women. This may indicate the presence of underlying concerns such as uncertainty about the rationale and safety of blood volumes obtained during clinical research that have been observed in other populations [31, 32, 51]. These concerns will need to be proactively explored and addressed to ensure the successful conduct of COVID-19 vaccine trials.

There are some limitations to our study. First, we did not use a particular theoretical framework in the selection of relevant predictor variables. We did however make use of existing literature on willingness-to-participate in HIV vaccine trials in our setting to develop the survey questionnaire. Second, we assessed hypothetical willingness-to-participate without reference to a specific trial or vaccination strategy. Previous studies have demonstrated large differences between hypothetical and actual willingness-to-participate in vaccine trials with the latter being much lower [48, 52]. Third, we conducted the study among HCWs in PNFP hospitals. Moreover, these HCWs had no direct experience in managing patients with COVID-19. Hence our findings may not be generalizable to all HCWs in Uganda particularly those with experience of managing COVID-19. It should be noted however, that the PNFP health sector is an integral part of Uganda’s healthcare system, employing about 35% of the country’s HCWs and accounting for more than 40% of the health sector outputs [53]. Also, management of COVID-19 in Uganda was restricted to a few select hospitals at the time of the study. Hence, majority of HCWs in the country would not have had experience in managing patients with COVID-19. Lastly, our findings may be susceptible to bias due to self-report. Key strengths of this study include the large sample size and thus improved precision around estimated parameters, and a high response rate (92%) which improves generalisability of findings. Although this study evaluated willingness-to-participate in COVID-19 vaccine trials, some of the findings e.g., concerns about the safety of vaccines, may be relevant to COVID-19 vaccine rollout programmes. Newman P et al demonstrated that concerns and motivators of willingness to participate in vaccine trials may extend to vaccines that have been approved [54].

In conclusion, willingness-to-participate in COVID-19 vaccine trials among HCWs in Uganda is high but may be affected by vaccine trial requirements and concerns about the safety of candidate vaccines. Preparation for COVID-19 vaccine trials should include extensive education about vaccine research and vaccine research requirements for potential participants.

## Supporting information

S1 Questionnaire (PDF)

S2 Questionnaire (PDF)
