# Supplementary material for: Willingness to participate in COVID-19 vaccine trials; a survey among a population of healthcare workers in Uganda
Source: PLoS One. 2021 May 27;16(5):e0251992. doi: 10.1371/journal.pone.0251992 (PMC8158909; doi:10.1371/journal.pone.0251992)
Supplement: S1 Questionnaire — (PDF) [file pone.0251992.s001.pdf]

COVID-19 VACCINE PREPAREDNESS (COVAP) STUDY QUESTIONAIRE

	Protocol code			Facility code		Participant ID				Date:	dd		MMM			yyyy			
**Study ID:**	C	V	P																

Demographics

1. Age (years)…………………

2. Gender *(mark only one)*
   a) Male
   b) Female

3. What is your marital status? *(mark only one)*
   a) Single (never married)
   b) Married/Cohabiting
   c) Divorced/separated/Widowed
   d) Prefer not to say

4. What is your occupation *(mark only one)*
   a) Medical doctor
   b) Clinical officer
   c) Nurse/Midwife
   d) Assistant nurse
   e) Radiology/X-ray technician
   f) Pharmacist/Pharmacy technician or dispenser
   g) Laboratory personnel
   h) Physical therapist
   i) Counselor
   j) Nutritionist/dietitian
   k) Student (medical/nursing/other)
   l) Administrator/Adminitrative assisant
   m) Other support staff (driver, cleaners, porters, guard, catering etc.)
   n) Other (specify):……………………

5. What is your highest level of education *(mark only one)*
   a) Primary school
   b) O-level
   c) A-level
   d) University
   e) Other higher institution
   f) None/never had any formal education

6. Number of persons living in your household (Youself included)

	Age group	Number
A	Children (<18 years)	
B	Adults (18-65)	
C	Older people (>65 years)	

COVID-19 VACCINE PREPAREDNESS (COVAP) STUDY QUESTIONAIRE

	Protocol code			Facility code		Participant ID				Date:	dd		MMM			yyyy			
**Study ID:**	C	V	P																

7. What are your sources of information on COVID-19. *(Mark all that apply)*
   a) Official international health organisation websites and media e.g. WHO, CDC.
   b) Official government websites and media e.g. Ministry of Health- Uganda.
   c) News Media e.g. TVs, radios, Magazines, Newspapers
   d) Social Media e.g. WhatsApp, Facebook, Twitter, Instagram
   e) Medical journals
   f) Others

Willingness to participate in COVID-19 vaccine research

8. Have you previously volunteered to participate in health research? *(mark only one)*
   a) Yes
   b) No

9. A number of clinical trials are being conducted in various parts of the world to test COVID-19 vaccines. Why must we do studies to test COVID-19 vaccines? *(circle all mentioned, don't read answers)*
   a) It is possible that COVID-19 vaccines may not work and must be tested first
   b) It is possible that COVID-19 vaccines may not be safe for people, and must be tested first
   c) There is not yet a vaccine to stop COVID-19
   d) Unsure
   e) Don't know
   f) Other (specify)……………………………………

10. Would you be willing to participate in COVID-19 vaccine trials? *(mark only one)*
   a) definitely yes
   b) probably yes
   c) definitely no
   d) probably no
   e) Not sure

11. If "definitely yes" or "probably yes", explain why *(Mark all that apply)*
   a) Altruism-It feels good to help the world find a vaccine
   b) Hope of being protected against getting COVID-19
   c) To get health care
   d) I may be paid some money for taking part
   e) Other (Specify)……………………………………………………………………

12. If "definitely no" or "probably no", explain why *(Mark all that apply and skip to question 42)*
   a) Anxiety/fear of catching novel coronavirus (SARS-CoV-2)
   b) Concerns over safety of vaccines
   c) Fear of injections

d) Fear of being stigmatized
e) Too busy/cannot commit time
f) Other (Specify)…………………………………………………………………………
g)

13. Is there any person you may need to consult before making a decision to participate in the COVID-19 vaccine trial? *(If not 'none' mark all that apply)*
a) None
b) Spouse
c) Parent/guardian
d) Other family member
e) Friend
f) Religious leader
g) Community leader
h) Healthcare worker
i) Other (Specify) …………………………………………………………………………

14. Would you be willing to participate in the COVID-19 vaccine trial if the trial schedule required to attend frequent (weekly to quarterly) clinic visits for safety monitoring over a 12-month period?
a) definitely yes
b) probably yes
c) definitely no
d) probably no
e) Not sure

15. Would you be willing to provide blood samples (approximately 50ml) for monitoring safety and immunogenicity of the vaccine at each of the clinic visits?
a) definitely yes
b) probably yes
c) definitely no
d) probably no
e) Not sure

Vaccine trial participants are usually allocated to one of two groups i.e. one that is injected with the experimental vaccine or one that is injected with a dummy product similar to the experimental vaccine but without the active ingredient in the vaccine (placebo)

16. Would you participate given the chance that you might receive either the candidate vaccine being tested or placebo?
a) definitely yes
b) probably yes
c) definitely no
d) probably no
e) Not sure

17. Vaccine injections may be associated with mild to moderate shortlived reactions at the injection site such as discomfort, warmth, redness and swelling and systemic symptoms such as fatigue, general malaise and headache. Would you participate given the risk of experiencing these reaactions?
    a) definitely yes
    b) probably yes
    c) definitely no
    d) probably no
    e) Not sure

During COVID-19 vaccine trials, pregnant women will be excluded from the study to protect the unborn child from any possible adverse effects that may be caused by the candidate vaccine. Men who are not sterilised will also be asked to avoid impregnating their female partners

18. **Women only:** Would you be willing to avoid/delay pregnancy by using effective contraception during the vaccine trial period?
    a) Incapable of becoming pregnant (menopause, sterile) / living a celebate life
    b) definitely yes
    c) probably yes
    d) definitely no
    e) probably no
    f) Not sure

19. **Men only:** Would you be willing to avoid impregnating your female partner during the trial?
    a) definitely yes
    b) probably yes
    c) definitely no
    d) probably no
    e) Not sure
    f) No partner
    g) I am incapable of making my partner pregnant (sterile)
    h) My partner is incapable of becoming pregnant e.g. menopause, sterile

Underlying health conditions

20. Do you have any underlying chronic health conditions? (Mark one only. If "No" or "Prefer not to say", stop here)
    a) Yes
    b) No
    c) Prefer not to say

21. If yes, do you have any of the following chronic health conditions
    a) Cardiovascular (Heart) disease, e.g. Hypertension
    b) Diabetes

	Protocol code			Facility code		Participant ID		
**Study ID:**	C	V	P					

Date: dd MMM yyyy

c) Asthma
d) Lung disease e.g. Tuberculosis
e) Liver disease
f) Sickle cell disease
g) HIV
h) Cancer
i) Obesity
j) Other (specify)………………………………………………..............
