# Supplementary material for: Willingness to participate in COVID-19 vaccine trials; a survey among a population of healthcare workers in Uganda
Source: PLoS One. 2021 May 27;16(5):e0251992. doi: 10.1371/journal.pone.0251992 (PMC8158909; doi:10.1371/journal.pone.0251992)
Supplement: S2 Questionnaire — (PDF) [file pone.0251992.s002.pdf]

COVID-19 VACCINE PREPAREDNESS STUDY (COVAP) QUESTIONAIRE

	Protocol code				Facility code			Participant ID				Date:									
**Study ID:**	C	V	P																		
													dd		MMM			yyyy			

Demographics Olina emyaka emeka? …………

1. Emyaka ………………………

2. Gender *(londako kimu)*
   a) Male
   b) Female

3. Oli mufumbo? *(Londako kimu)*
   a) Siri mufumbo (ssiwasangako/ssifumbirwangako)
   b) Ndi mufumbo/Nina omuntu gwetuberagana naye
   c) Twayawukana/Omwagalwa wange yafa
   d) Ssaagala kukiddamu

4. Okola mulimu ki? *(Londako gumu)*
   a) Medical doctor
   b) Clinical officer
   c) Nurse/Midwife
   d) Assistant nurse
   e) Radiology/X-ray technician
   f) Pharmacist/Pharmacy technician or dispenser
   g) Laboratory personnel
   h) Physical therapist
   i) Counselor
   j) Nutritionist/dietitian
   k) Student (medical/nursing/other)
   l) Administrator/Adminitrative assisant
   m) Other support staff (driver, cleaners, porters, guard, catering etc.)
   n) Other (specify):……………………

5. Mu by'obuyigirize, wakoma ku ddaala ki?*(Londako kimu)*
   a) Primary school
   b) O-level
   c) A-level
   d) University
   e) Other higher institution
   f) Ssaasomako

6. Amakaago mulimu abantu bameka? (Nga naawe weebaliddemu)

	Ekibinja ky'emyaka	Omuwendo
A	Abaana abataweza myaka18)	
B	Abakulu (emyaka 18-65)	
C	Abakadde (abasussa emyaka 65)	

COVID-19 VACCINE PREPAREDNESS STUDY (COVAP) QUESTIONAIRE

Study ID:	Protocol code	Facility code	Participant ID	Date:	dd	MMM	yyyy
	C V P						

7. Ebikwata ku bulwadde bwa COVID-19 obifuna kuva mu makubo ki? *(londako byonna ebituufu)*
   a) Official international health organisation websites and media e.g. WHO, CDC.
   b) Official government websites and media e.g. Ministry of Health- Uganda.
   c) News Media e.g. TVs, radios, Magazines, Newspapers
   d) Social Media e.g. WhatsApp, Facebook, Twitter, Instagram
   e) Medical journals
   f) Others (specify)…………………………………………………………………..

Willingness to participate in COVID-19 vaccine research

8. Wali wetabyeko mu kunonyereza kw'eby'obulamu? *(londako kimu kyokka)*
   a) Yee
   b) Nedda

9. Okunoonyereza okuwerako kugenda mu maaso munsi yonna okugezesa eddagala ly'okugema COVID-19. Lwaki tuteekeddwa okukola okunoonyereza okugezesa eddagala ly'okugema COVID-19? *(laga byonna byayogedde)*
   a) Kisoboka eddagala ly'okugema obutakola,era litekeddwa okusooka okugezesebwa.
   b) Kisoboka eddaga obutaba ddungi eri abantu, era litekeddwa okusooka okugezesebwa
   c) Tewannazulibwa ddagala liziyiza COVID-19
   d) Sseekakasa lwaki
   e) Ssimanyi
   f) Ensonga endala (nnyonnyola)……………………………………

10. Wandibadde mwetegefu okwetaba mu kunoonyereza kw'okugezesa eddagala ly'okugema COVID-19 *(londako kimu)*
    a) Tewali kubuusabuusa ndi mwetegefu
    b) Oba olyawo nyinza
    c) Nedda, siri mwetegefu kwetabamu nnaakamu
    d) Oba olyawo nyiza obutetabaamu
    e) Sseekakasa

11. Bwe kiba nti olonzeewo "tewali kubuusabuusa oli mwetegefu"; Oba "oba olyawo oyinza" okwetabamu nnyonyola, lwaki? *(laga byonna byayogedde))*
    a) Kyakwenyumiriza okuyamba ensi okuzuula eddagala
    b) Essuubi ly'okufuna obukuumi eri ekirwadde kya COVID-19
    c) Okufuna obujjanjabi obulala
    d) Nkyayinza okusasulwayo ku ssente olw'okwetabamu
    e) Ensonga endala (Nnyonnyola)…………………………………………………………………

12. Bw'oba walonzeewo "mu buli ngeri nedda" oba "osanga nedda", nnyonnyola lwaki *(laga byonna byayogedde), ogende ku kibuuzo ekya 41)*

Study ID:	Protocol code: C V P	Facility code:	Participant ID:	Date:	dd	MMM	yyyy

a) Okutya/okwelaliikirira okukwatibwa akawuka akaleeta COVID-19 (SARS-CoV-2)
b) Okwelarikilira obulabe obuyinza okuva mu ddagala erigema
c) Okutya empiso
d) Okutya okusosolwa
e) Nnina eby'okukola bingi/Ssiyinza kuwaawo budde kwetabamu
f) Ensonga endala (nnyonnyola)……………………………………………………

13. Waliwo omuntu gw'oyinza okwetaaga okwebuzaako nga tonnasalawo kwetaba mu kunonyereza kw'okugezesa eddagala ly'okugema COVID-19? *(laga byonna byayogedde))*
a) Tewali
b) Omwagalwa wange
c) Omuzadde/Oyo aninako obuvunaanyizibwa
d) Ow'omu maka gaffe omulala
e) Mukwano gwange
f) Omukulembeze w'eddini
g) Omukulembeze mu kitundu
h) Omusawo
i) Omulala yenna, (mwogere) ……………………………………………………

14. Wandibadde mwetegefu okwetaba mu kunoonyereza kw'okugezesa eddagala ly'okugema COVID-19 singa wesanga nga kikwetaagisa okugenda ku kitebe ky'okunoonyereza emirundi egiwerako (buli wiiki ne buli luvanyuma lwa myezi esatu) mu bbanga lya myezi 12?
a) Yee ndi mwetefu ddala
b) Oba lyawo ndi mwetegefu
c) Nedda, siri mwetegefu nnaakamu
d) Oba olyawo siri mwetegefu
e) Sseekakasa

15. Wandibadde mwetegefu okuwaayo omusaayi (nga 50mls) buli lw'ojja ku kitebe ky'okunoonyereza, okukeberebwa okulondoola nti eddagala teririna bulabe ate n'okulaba oba liyamba omubiri okukola abaserikale?
a) Tewali kubuusabuusa ndi mwetegefu
b) Oba olyawo nyinza okuba omwetegefu
c) Nedda, siri mwetegefu nnaakamu
d) Oba olyawo siri mwetegefu
e) Sseekakasa

Mu nkola eya bulijjo, abantu abeetaba mu kunoonyereza kw'okugezesa eddagala ly'okugema bateekebwa mu kimu ku bibinja ebibiri; abali mu kibinja ekimu bafuna obuyiso bw'eddagala erigezesebwa, ate abalala nebatekebwa mu kibinja ekifuna obuyiso omuli ekintu ekyefaanaanyiriza eddgala erigezesebwa naye nga mukyo temuli kirungo kya ddagala. Ekintu kino kiyitibwa "Placebo" mu lulimi ly'okunoonyereza.

Study ID:	Protocol code	Facility code	Participant ID	Date:	dd	MMM	yyyy
	C V P						

16. Wandyetabyemu bwekiba nga okuyita mu nkola ey'akalulu etaliimu kyekubiira, okyayinza okufuna eddagala erigezesebwa lyennyini oba ekyo omutali kirungo?
    a) Tewali kubuusabuusa ndi mwetegefu
    b) Oba olyawo nyinza okuba omwetegefu
    c) Nedda, siri mwetegefu nnaakamu
    d) Oba olyawo siri mwetegefu
    e) Sseekakasa

17. Empiso z'okugema ziyinza okuleeta obuzibu obutonono awo empiso wekubiddwa naye nga obuzibu buno buggwawo mu bbanga ttono ddala. Muno muyinza okubaamu okuwulira obubi, ebbugumu, okumyukirira, n'okuzimba, wamu n'obubonero obulala nga; okuwulira obukowukoowu, okwewulira nga tolina maanyi bulungi, n'okulumizibwa omutwe. Wandyetabyemu ng'otegedde obuzibu buno bw'oyinza okufuna?
    a) Tewali kubuusabuusa ndi mwetegefu
    b) Oba olyawo nyinza okuba omwetegefu
    c) Nedda, siri mwetegefu nnaakamu
    d) Oba olyawo siri mwetegefu
    e) Sseekakasa

Nga okunoonyereza kw'okugezesa eddagala ly'okugema COVID-19 kukolebwa, abakyala abali embuto tebajja kwetabamu olw'okwerinda obuzibu eddagala bwe liyinza okutuusa ku bulamu bw'omwana ali mu lubuto. Abaami abataasalibwa buseke okubataangira okufunyisa olubuto nabo bajja kusabibwa okuziyiza okufunyisa abagalwa babwe olubuto/embuto.

18. **Bakyala bokka:** Wandisobodde okweziyiza/okwongezaayo okufuna olubuto ng'okozesa enkola eya kizaala gumba mu kiseera ky'onoonyereza kw'okugezesa eddagala?
    a) Ssikyazaala (ndi mu myaka egitakyazaala, bankomya)
    b) Tewali kubuusabuusa ndi mwetegefu mu buli ngeri ye
    c) Oba olyawo nyinza okuba omwetegefu
    d) Nedda, ssiri mwetegefu nnaakamu mu buli ngeri nedda
    e) Oba olyawo siri mwetegefu osanga nedda
    f) Sseekakasa

19. **Abasajja bokka:** Wandikkiriza okwewala okufunyisa omwagalwa wo olubuto nga okunoonyereza kugenda mu maaso?

    a) Tewali kubuusabuusa ndi mwetegefu
    b) Oba olyawo nyinza okuba omwetegefu osanga ye
    c) Nedda, ssiri mwetegefu nnaakamu mu buli ngeri nedda
    d) Oba olyawo siri mwetegefu osanga nedda
    e) Sseekakasa
    f) Ssirina mubeezi/gwentabagana naye mu bikolwa eby'ekyaama
    g) Ssisobola kufunyisa mwagalwa wange lubuto (bankomya)
    h) Omwagalwa wange tasobola kufuna lubuto, okugeza, takyazaala (ali mu myaka egitazaala, bamukomya)

COVID-19 VACCINE PREPAREDNESS STUDY (COVAP) QUESTIONAIRE

	Protocol code			Facility code		Participant ID			Date:	dd		MMM			yyyy			
**Study ID:**	C	V	P															

Underlying health conditions

20. Olina obulwadde obw'oluteentezi bwonna?
   a) Yee
   b) Nedda
   c) Ssandyagadde kukyogerako

21. Bwekiba nti "yee" olina obumu kubulwadde buno wammanga?
   a) Obulwadde bw'omutima, okugeza,puleesa
   b) Ssukaari
   c) Asima
   d) Obulwadde bw'amawugwe, okugeza, akafuba
   e) Obulwadde bw'ekibumba
   f) Obulwadde bwa nnalubiri
   g) Akawuka akaleeta mukenenya
   h) Kaansa
   i) Omugejjo oguyitiridde
   j) Obulala (nnyonnyola)_______________________________
